# Clinical Research on Lysergic Acid Diethylamide (LSD) in Psychiatry and Neuroscience

**DOI:** 10.3390/ph18040499

**Published:** 2025-03-29

**Authors:** Hossein Omidian, Alborz Omidian

**Affiliations:** 1 Barry and Judy Silverman College of Pharmacy, Nova Southeastern University, Fort Lauderdale, FL 33328, USA; 2 Department of Psychiatry, Westchester Medical Center, Valhalla, NY 10595, USA; alborz.omidian@wmchealth.org

**Keywords:** LSD, psychedelics, serotonin receptors, pharmacogenetics, clinical research

## Abstract

Lysergic acid diethylamide (LSD) is gaining renewed interest as a potential treatment for anxiety, depression, and alcohol use disorder, with clinical trials reporting significant symptom reductions and long-lasting effects. LSD modulates serotonin (5-HT2A) receptors, which, in turn, influence dysfunctional brain networks involved in emotional processing and cognition. It has also shown promise in psychedelic-assisted psychotherapy, where mystical-type experiences are linked to improved psychological well-being. This review examines LSD’s pharmacokinetics, neurobiological mechanisms, and safety considerations, including cardiovascular risks, emotional vulnerability, and the potential for hallucinogen-persisting perception disorder (HPPD). Challenges such as small sample sizes, variable dosing protocols, and regulatory restrictions limit large-scale trials. Future research should focus on standardization, pharmacogenetic influences, and personalized treatment strategies to ensure its safe and effective integration into clinical practice.

## 1. Introduction

Lysergic acid diethylamide (LSD) was first synthesized in 1938 by Swiss chemist Albert Hofmann at Sandoz Pharmaceuticals in Basel, Switzerland [1]. Initially studied as part of research into ergot alkaloids (*Claviceps purpurea*) for pharmaceutical development, LSD showed no significant pharmacological effects at the time. However, in 1943, Hofmann revisited the compound and serendipitously discovered its potent psychedelic properties [2]. On 19 April 1943, he intentionally ingested a small dose and experienced its profound psychoactive effects, marking what is now celebrated as Bicycle Day—the first recorded LSD trip [3].

Following its discovery, LSD attracted significant scientific interest, particularly in psychiatry and neuroscience, due to its ability to induce altered states of consciousness. In the 1950s and 1960s, researchers explored its therapeutic potential for conditions such as alcoholism, depression, and other psychiatric disorders [4]. However, its widespread recreational use and association with countercultural movements led to increasing governmental restrictions. Despite these limitations, modern research continues to investigate LSD’s potential in treating depression, anxiety, and post-traumatic stress disorder (PTSD) [5].

Advancements in organic chemistry have facilitated a concise synthesis of lysergic acid from simple aromatic precursors using a six-step process from commercially available materials. This strategy—employing coupling, dearomatization, and cyclization—has enabled the development of novel neuroactive compounds, including 12-chlorolysergic acid, a newly identified natural product derivative [6].

Mid-20th-century regulatory changes profoundly affected LSD research. The Kefauver Harris Drug Amendments of 1962 imposed stricter requirements, mandating that all drugs demonstrate efficacy through controlled clinical trials before approval. These changes posed significant challenges for LSD psychotherapy, as its subjective and experiential effects proved difficult to evaluate through traditional trial methodologies. Additionally, rising public concerns over its recreational use led to the suspension of clinical research for decades [7,8].

In recent years, renewed interest in psychedelic science has reestablished LSD as a subject of clinical investigation. Advances in neuroscience and psychopharmacology have shed light on its pharmacological profile, particularly its high-affinity agonist activity at serotonin (5-HT) receptors, most notably the 5-HT_2_A receptor [9,10]. Neuroimaging studies reveal that LSD significantly alters functional connectivity within brain networks [11,12]. Unlike conventional pharmacotherapies, its effects are highly dose-dependent and influenced by psychological and environmental factors, commonly known as “set and setting” [13,14].

Despite its promise, LSD research remains in its early stages, with several methodological challenges. Studies on its therapeutic applications are constrained by small sample sizes and inconsistent dosing protocols [15,16,17,18]. While some studies are conducted in clinical settings, others rely on self-reported data from nonclinical populations, complicating standardization efforts [19]. Regulatory barriers stemming from its Schedule I classification continue to hinder large-scale trials and broader clinical acceptance [7,8]. Moreover, individual variability in LSD’s effects, potentially influenced by pharmacogenetics and serotonin receptor activity, highlights the need for personalized treatment approaches [9,10].

As research progresses, addressing these limitations will be crucial to establishing LSD’s role in modern medicine. This review synthesizes the current state of clinical LSD research, examining its pharmacokinetics, neurobiological mechanisms, safety considerations, and therapeutic applications. By identifying critical gaps and outlining future directions, this analysis aims to support the development of evidence-based psychedelic therapies. Integrating interdisciplinary findings while overcoming regulatory and methodological challenges will be key to advancing the scientific understanding of LSD’s potential in clinical practice.

## 2. Pharmacology and Mechanistic Foundations of LSD

### 2.1. LSD Metabolism and Pharmacokinetics

LSD is rapidly absorbed following oral administration, with peak plasma concentrations typically reached within 1.2 to 2 h [17,20,21]. The drug exhibits first-order elimination kinetics and demonstrates a biphasic elimination pattern. Reported elimination half-lives range between 3.6 and 8.9 h, depending on the study population and dosage regimen [20,21]. The subjective effects of LSD generally persist for 8 to 12 h, aligning with its pharmacokinetic profile [21,22].

**Figure 1:**
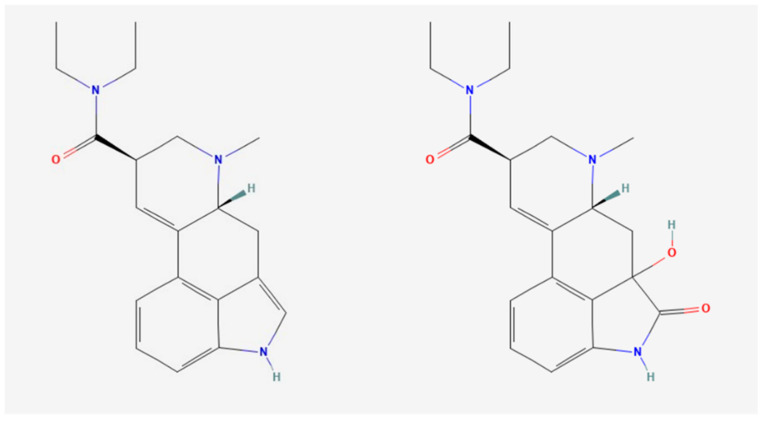
Chemical structures of lysergic acid diethylamide (LSD) and its main metabolite 2-Oxo-3-hydroxy-lysergide [24,25].

LSD undergoes extensive hepatic metabolism primarily via cytochrome P450 enzymes, including CYP2D6, CYP3A4, and CYP1A2. These enzymes contribute to the formation of principal metabolites such as nor-LSD and 2-oxo-3-hydroxy-LSD [23]. Nor-LSD retains some hallucinogenic activity, whereas 2-oxo-3-hydroxy-LSD is considered pharmacologically inactive. In vitro investigations further identified CYP2E1, CYP2C9, and CYP1A2 as contributors to the biotransformation of LSD [23]. These metabolic pathways are clinically relevant, as genetic polymorphisms or pharmacological interactions affecting these enzymes could influence individual variability in LSD metabolism and its associated effects. Figure 1 illustrates the chemical structures of LSD and its major metabolite, highlighting the key modifications that occur during metabolism [24,25].

Clinical pharmacokinetic studies have provided detailed insights into LSD absorption, distribution, metabolism, and excretion. In one study involving 16 healthy participants, the oral administration of 200 µg LSD resulted in a mean peak plasma concentration of 4.5 ± 1.4 ng/mL at approximately 1.5 h post dose. Plasma concentration curves exhibited a biphasic decline, with terminal half-lives of 3.6 and 8.9 h. No significant sex-based differences in pharmacokinetics were observed. Subjective effects strongly correlated with plasma levels and lasted up to 12 h [20].

A dose-proportional relationship was confirmed in a subsequent comparative study. The oral administration of 100 µg and 200 µg LSD produced plasma concentrations proportional to the dose, with no evidence of acute tolerance during the observation period. Subjective effects remained consistent in duration and intensity across both dosing regimens, lasting between 8 and 12 h [21].

The advancement of analytical methodologies has significantly enhanced the detection and quantification of LSD and its metabolites. Liquid chromatography–tandem mass spectrometry (LC-MS/MS) has been validated for the measurement of LSD, iso-LSD, O-H-LSD, and nor-LSD in plasma. While LSD was quantifiable in all samples, metabolite concentrations were generally below the limit of detection [26]. These methods provide high precision and accuracy, supporting their application in both clinical and forensic contexts. Additionally, ultra-high-performance liquid chromatography with tandem mass spectrometry (UHPLC-MS/MS) demonstrated that the use of sodium fluoride (NaF) minimizes LSD degradation in biological samples. Detection limits as low as 0.5 pg/mL have been achieved, allowing for the trace-level identification of LSD and its analogs [27].

Postmortem toxicological investigations have reported median LSD blood concentrations of approximately 0.8 µg/L. In such cases, severe agitation was the most frequently observed clinical presentation, occurring in 27.3% of instances [28]. While LSD is not commonly associated with direct toxicity, individual variability in metabolic enzyme function or drug interactions could influence clinical outcomes. Moreover, the identification of LSD analogs such as 1CP-LSD, which act as prodrugs, underscores the role of metabolic conversion in modulating both the intensity and duration of LSD’s effects [29,30].

### 2.2. Receptor Binding and Physiological Effects

LSD primarily exerts its psychoactive effects through agonism at the serotonin 5-HT_2_A receptor, which is central to its hallucinogenic and psychedelic properties. In addition to 5-HT_2_A, LSD interacts with several other serotonergic receptors, including 5-HT_1_A, 5-HT_2_B, and 5-HT_2_C, as well as dopaminergic and adrenergic receptors [23,31]. Notably, LSD binds to 5-HT_2_A receptors in a pseudo-irreversible manner, which is hypothesized to contribute to its prolonged psychoactive duration compared to other serotonergic psychedelics such as DOI [31].

This extended receptor interaction may partially account for the sustained alterations in cognition and perception observed following LSD administration. Radioligand binding studies using [^125^I]LSD and [^125^I]DOI have demonstrated dense receptor occupancy in brain regions including the cortex, nucleus accumbens, and cingulate gyrus, consistent with LSD’s serotonergic activity [32]. These findings are relevant for neuroimaging research, particularly in the context of mapping serotonin receptor distributions using techniques such as positron emission tomography (PET).

Beyond serotonergic effects, LSD influences physiological parameters through the modulation of endocrine and autonomic systems. It has been shown to significantly elevate the plasma levels of cortisol, prolactin, oxytocin, and epinephrine, resulting in increased heart rate, blood pressure, and body temperature [33]. In studies involving doses of 100 µg or greater, moderate increases in the peak heart rate (exceeding 100 bpm) were observed in approximately 20–25% of participants. Similarly, body temperatures exceeding 38 °C were reported in up to 34% of cases [18]. These findings suggest a need for medical screening and careful dose titration, particularly in individuals with pre-existing cardiovascular conditions.

The role of 5-HT_2_A receptors extends to LSD-induced oxytocin release, which has been associated with the drug’s prosocial and empathogenic effects [10]. Although these effects may have therapeutic relevance, they also raise safety considerations, such as increased emotional sensitivity and suggestibility during the LSD experience. These psychological states underscore the importance of controlled and supportive settings for clinical or experimental use.

Animal studies have reinforced the role of serotonergic and dopaminergic systems in mediating LSD’s behavioral effects. For example, in rabbit models, both LSD and DOI induced head-bobbing behavior through 5-HT_2_A and dopamine D_1_ receptor activation. While LSD displayed moderate affinity for the D_1_ receptor, DOI exhibited minimal interaction, suggesting that dopaminergic pathways may complement serotonergic mechanisms in producing LSD’s characteristic effects [31].

Recent preclinical research has also focused on novel LSD analogs. One such compound, 1CP-LSD, has been identified as a prodrug that converts to LSD in human serum. Analytical studies utilizing mass spectrometry, chromatography, and nuclear magnetic resonance (NMR) confirmed this bioconversion. Behavioral assays in mice demonstrated LSD-like activity, with a median effective dose (ED_50_) of 430.0 nmol/kg, closely aligning with 1P-LSD (ED_50_ = 349.6 nmol/kg) [29].

Conversely, 1-dodecanoyl-LSD (1DD-LSD), another emerging lysergamide, exhibited significantly lower potency in head-twitch response (HTR) assays—approximately 27 times less potent than LSD. This reduced efficacy is likely due to the inefficient in vivo hydrolysis of its dodecanoyl moiety. While its enhanced lipophilicity may affect its pharmacokinetic behavior, further research is needed to assess its potential clinical or recreational applications [34].

### 2.3. Neurophysiological and Brain Connectivity

LSD’s effects on brain function and connectivity have been extensively characterized through advanced neuroimaging studies, which provide mechanistic insights into its psychoactive and potential therapeutic properties. Functional magnetic resonance imaging (fMRI) studies have consistently demonstrated that LSD induces widespread alterations in brain network dynamics.

In a study involving 20 healthy participants, LSD significantly decreased functional connectivity within intrinsic brain networks, including the visual, sensorimotor, auditory, and default mode networks. Simultaneously, it enhanced global connectivity between distinct brain regions, particularly between subcortical structures (thalamus, striatum) and cortical hubs such as the precuneus and anterior cingulate cortex [11]. These findings suggest a reorganization of neural communication under LSD, which may underlie its perceptual distortions and altered cognitive states.

Further supporting this, a double-blind, randomized, placebo-controlled fMRI study involving 25 participants employed regression dynamic causal modeling (rDCM) to examine LSD’s effects on cortico-striato-thalamo-cortical (CSTC) circuits. LSD increased connectivity from the thalamus to the posterior cingulate cortex via serotonin 5-HT_2_A receptor activation while simultaneously decreasing connectivity from the ventral striatum to the thalamus [12]. These results are consistent with the thalamic filter model of psychedelic action, which posits that psychedelics disrupt sensory gating, leading to an influx of sensory and cognitive information that may contribute to altered states of consciousness.

The computational modeling of fMRI data has provided further evidence of LSD’s impact on global brain dynamics. One study demonstrated that LSD increased both perturbational sensitivity and response diversity, particularly within the limbic, visual, and default mode networks. This suggests that LSD shifts the brain into a more flexible, less constrained state, potentially enhancing responsiveness to internal and external stimuli [35].

In addition to sensory and cognitive effects, LSD influences social cognition and emotional processing. A placebo-controlled crossover study (N = 16) investigated the effects of LSD on emotional empathy and neurohormonal responses. LSD dose-dependently increased emotional empathy, with the 200 µg dose also elevating plasma oxytocin levels. Interestingly, pretreatment with the 5-HT_2_A receptor antagonist ketanserin blocked the oxytocin response but did not fully suppress empathy enhancement, suggesting that LSD’s prosocial effects may involve receptor systems beyond 5-HT_2_A [10].

These changes in connectivity and emotional processing have implications for psychiatric treatment. LSD has been associated with enhanced emotional empathy and the disruption of rigid cognitive patterns, which may benefit individuals with conditions such as depression, anxiety, PTSD, and addiction [10,11,12]. By modulating brain networks implicated in self-referential thinking and emotional regulation, LSD may help shift maladaptive patterns of thought and behavior.

Importantly, LSD’s dose–response relationship follows a sigmoid-like curve, with perceptual and physiological effects plateauing at approximately 100 µg. Doses above this threshold do not necessarily increase therapeutic efficacy but may elevate the risk of adverse effects, highlighting the need for careful dose optimization in clinical applications [13].

### 2.4. Neuroplasticity and Biomarkers

Emerging evidence suggests that LSD may influence neuroplasticity through the modulation of specific molecular and cellular pathways. Neuroplasticity, the brain’s capacity to reorganize and form new synaptic connections, is critically involved in learning, memory, and recovery from psychiatric disorders. One of the key biomarkers of neuroplasticity is brain-derived neurotrophic factor (BDNF), a protein that supports the growth, survival, and differentiation of neurons.

A placebo-controlled study in healthy human participants demonstrated that low doses of LSD (5, 10, and 20 µg) significantly increased plasma BDNF levels. These elevations were observed approximately 4–6 h after administration, suggesting an acute effect of LSD on neurotrophic signaling [36]. This study provides preliminary evidence that LSD may promote neuroplasticity in humans, although further research is required to determine whether these molecular changes translate into clinically meaningful outcomes, particularly in the context of psychiatric treatment.

Complementary findings have been reported in preclinical models. In a study conducted in male mice, the repeated—but not acute—administration of LSD (30 µg/kg, daily for seven days) resulted in enhanced social interaction behaviors. Mechanistic investigations revealed that this prosocial effect was mediated through the activation of serotonin 5-HT_2_A and AMPA receptors in the medial prefrontal cortex, and it was dependent on mammalian target of rapamycin complex 1 (mTORC1) signaling within excitatory neurons [37]. These results indicate that LSD facilitates excitatory synaptic transmission and supports the recruitment of neuroplasticity-related pathways.

Together, human and animal studies suggest that LSD may exert beneficial effects on neuroplasticity, possibly contributing to its reported prosocial and cognitive-enhancing properties. The involvement of BDNF, AMPA receptors, and mTORC1 signaling highlights specific molecular targets through which LSD may modulate synaptic function and behavioral outcomes. These findings underscore the importance of continued research to clarify the long-term implications of LSD-induced plasticity and its relevance to therapeutic interventions.

### 2.5. Genetic Polymorphisms in ADME and Receptor Activity

Interindividual variability in response to LSD may be influenced by genetic polymorphisms that affect its absorption, distribution, metabolism, and excretion (ADME), as well as its interaction with central nervous system receptors. Understanding these genetic factors is critical for optimizing safety, efficacy, and personalized treatment approaches in psychedelic-assisted therapy.

#### 2.5.1. Cytochrome P450 Polymorphisms and LSD Metabolism

LSD is metabolized primarily by hepatic cytochrome P450 (CYP) enzymes, including CYP2D6, CYP3A4, and CYP1A2. These enzymes facilitate the biotransformation of LSD into key metabolites such as nor-LSD and 2-oxo-3-hydroxy-LSD (O-H-LSD) [23]. Genetic polymorphisms in these isoenzymes can significantly affect metabolic capacity, contributing to variations in drug clearance, duration of action, and subjective effects.

CYP2D6, in particular, is highly polymorphic, with phenotypes classified into poor, intermediate, extensive, and ultra-rapid metabolizers. Individuals with reduced CYP2D6 activity may exhibit slower LSD clearance, potentially resulting in prolonged or intensified effects. In contrast, ultra-rapid metabolizers could eliminate the drug more quickly, leading to a reduced pharmacodynamic response. Similarly, polymorphisms in CYP3A4 and CYP1A2 have been associated with altered drug metabolism, which could also affect LSD’s systemic exposure and duration of action. As these enzymes collectively contribute to LSD metabolism, pharmacogenetic screening may offer predictive value for tailoring individual dosing regimens in clinical settings.

#### 2.5.2. Serotonin Receptor Genetic Variability and LSD Response

LSD’s psychoactive effects are mediated primarily through agonism at the serotonin 5-HT_2_A receptor, but additional interactions with 5-HT_1_A, 5-HT_2_B, and 5-HT_2_C receptors also contribute to its overall profile [23]. Genetic variation in serotonin receptor genes, particularly HTR2A, which encodes the 5-HT_2_A receptor, has been associated with differential sensitivity to psychedelic compounds.

Polymorphisms in HTR2A have been linked to variability in subjective psychedelic experiences, risk for psychiatric disorders such as schizophrenia, and response to serotonergic antidepressants. These genetic differences may contribute to individual differences in the intensity or character of LSD-induced perceptual and cognitive alterations. Additionally, genetic variability in serotonin receptor expression has been implicated in treatment response among individuals with social anxiety disorder, further supporting a role for serotonergic gene polymorphisms in modulating drug effects [38].

Developmental and functional changes in serotonin receptor expression linked to genetic variation may also affect vegetative and autonomic functions. Such alterations could influence physiological responses to LSD, including cardiovascular and thermoregulatory effects, which are mediated in part by serotonergic signaling [39]. These findings underscore the importance of receptor-level genetic diversity in shaping both the experiential and physiological outcomes of LSD use.

#### 2.5.3. Genetic Influence on Drug Responsiveness and Psychopharmacology

In addition to metabolism and receptor activation, other genetic factors may contribute to individual differences in responsiveness to LSD and related compounds. For instance, variability in LSD-displacing factors (LDFs)—endogenous molecules that may influence drug binding or availability—has been proposed to affect individual sensitivity to antipsychotic medications [40]. Although the precise role of LDFs in LSD pharmacology remains unclear, their potential involvement suggests another layer of genetic modulation in psychopharmacological responses.

**Figure 2:**
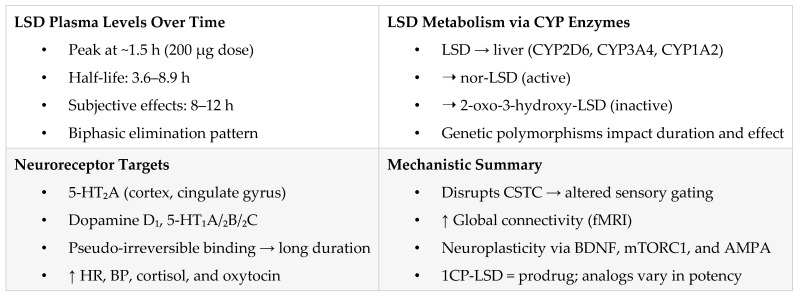
Pharmacology and mechanistic foundations of LSD [11,20,23,31,36].

Figure 2 summarizes LSD’s absorption, metabolism, receptor activity, and neuroplastic effects, highlights key enzymes (CYPs), 5-HT_2_A binding, and analog pharmacology (1CP-LSD, 1DD-LSD), and connects pharmacokinetics with subjective and physiological responses.

## 3. Therapeutic Applications of LSD

The therapeutic use of LSD has re-emerged as an area of serious scientific inquiry. Research spans full-dose psychedelic therapy, microdosing, pain management, and cognitive enhancement. Clinical interest is driven by LSD’s potential to address treatment-resistant psychiatric conditions, substance use disorders, and chronic pain. However, the findings remain preliminary in many areas, and further validation is needed through well-controlled, large-scale studies.

### 3.1. LSD in Psychotherapy

LSD-assisted psychotherapy has shown promise in promoting psychological insight, emotional release, and symptom relief when administered in structured, medically supervised settings. A Swiss observational study investigating LSD- and 3,4-methylenedioxymethamphetamine (MDMA)-assisted psychotherapy for patients with major depression and PTSD found that LSD doses of 100–200 µg induced pronounced alterations of consciousness, including mystical-type experiences, as measured by the Mystical Experience Questionnaire. These acute subjective effects, which were comparable to those observed in clinical trials and among healthy volunteers, were associated with therapeutic benefit in the context of group-based, drug-assisted psychotherapy delivered under a compassionate use program [41].

Historical clinical data also provide relevant context for understanding long-term treatment effects. A follow-up study of twenty psychiatric patients treated with a single large dose of LSD found that those who experienced an immediate, insight-driven response showed greater early symptom improvement. However, this group also exhibited a higher tendency to relapse within six months. In contrast, patients with slower initial responses often demonstrated progressive improvement between six and twelve months post treatment. The most favorable outcomes were observed in patients with specific conduct disorders and relatively well-developed personality structures, suggesting the importance of individual psychological profiles in predicting therapeutic response. Notably, patients in the LSD-treated group showed greater improvement overall compared to a matched control group receiving psychotherapy without LSD, despite a lower frequency of psychotherapeutic support in the LSD group [42]. These findings underscore the therapeutic potential of LSD-assisted psychotherapy while emphasizing the necessity for careful patient selection, individualized treatment planning, and professional oversight.

### 3.2. LSD for Anxiety, Depression, and End-of-Life Distress

LSD has been explored for its anxiolytic and antidepressant effects, particularly in individuals with treatment-resistant psychiatric conditions and those facing life-threatening illnesses. In a large-scale Phase IIb trial involving 198 participants, a single 100 µg dose of LSD resulted in symptom remission in 50% of individuals diagnosed with generalized anxiety disorder (GAD) [43]. Additionally, clinical trials assessing LSD for anxiety and depression in the context of terminal illness found that 77% of participants experienced durable relief from anxiety symptoms one year after treatment [43].

These findings are supported by a meta-analysis of randomized controlled trials from the mid-20th century, which reported that a single dose of LSD was significantly associated with reductions in alcohol misuse, with an odds ratio of 1.96 (*p* = 0.0003) [44]. While the results underscore LSD’s potential to deliver rapid and lasting symptom relief, particularly in emotionally burdensome conditions, the therapeutic outcomes are highly contingent on set (the patient’s mindset), setting (the therapeutic environment), and individual variability. These factors necessitate rigorous clinical protocols, thoughtful patient selection, and structured integration support in modern applications.

### 3.3. Alcohol Use Disorder (AUD)

Among LSD’s earliest therapeutic applications is the treatment of AUD. A meta-analysis of six randomized controlled trials (N = 536) found that a single dose of LSD was significantly associated with reduced alcohol misuse (OR = 1.96; 95% CI: 1.36–2.84; *p* = 0.0003), with no evidence of between-study heterogeneity (I^2^ = 0%) [44]. Early LSD interventions in the 1950s and 1960s, particularly those conducted in Saskatchewan, Canada, demonstrated notable abstinence rates in alcohol-dependent patients and were supported by provincial authorities, Alcoholics Anonymous chapters, and public health organizations [7]. However, critics questioned the methodological rigor of these studies, which often lacked appropriate controls, limiting their influence on broader medical practice.

The 1962 Kefauver Harris Drug Amendments in the U.S. further constrained psychedelic research by requiring that drug efficacy be established through well-controlled clinical trials. This regulatory shift posed a challenge to LSD psychotherapy, which depended on subjective, individualized experiences that were difficult to standardize within conventional trial designs [8].

In contemporary research, preclinical studies in animal models have cast doubt on the consistency of psychedelics in treating AUD. For example, a 2020 study using the alcohol deprivation effect (ADE) model in rats found that neither high-dose nor microdose LSD produced sustained anti-relapse effects, highlighting the need for more translational human research to evaluate real-world therapeutic potential [45].

### 3.4. Microdosing and Mood Disorders

Microdosing—defined as the repeated intake of sub-hallucinogenic doses of psychedelics—has attracted increasing interest for its potential in treating depression and enhancing cognition. In an open-label pilot trial (LSDDEP1) involving 20 patients with major depressive disorder (MDD), participants received 5–15 µg doses of LSD twice weekly for eight weeks. The study reported high adherence and good tolerability but did not establish antidepressant efficacy [16]. A subsequent Phase 2b randomized controlled trial (LSDDEP2) is now underway to evaluate depressive symptom reduction, EEG and biomarker changes, and safety using titrated doses of 4–20 µg administered twice weekly in a naturalistic home setting [46].

In a separate six-week randomized controlled trial involving 80 healthy adult males, participants received 14 doses of 10 µg LSD or placebo every three days. While the LSD group reported acute mood-elevating effects on dose days—including increased creativity, connectedness, and happiness—no sustained improvements in mood or cognition were observed at the six-week endpoint. Anxiety emerged as the most significant adverse effect, leading to the withdrawal of four participants from the LSD group [19].

Another finding from this trial was that microdosing significantly increased sleep duration the night after dosing. Participants in the LSD group slept an average of 24.3 min longer than those in the placebo group, without changes in sleep architecture or daytime activity levels [15]. These results indicate potential physiological benefits, though the clinical relevance remains to be established.

Taken together, the current evidence suggests that LSD microdosing is generally well tolerated, but its therapeutic efficacy for mood disorders remains unproven. Further placebo-controlled studies with optimized dose titration and active placebo controls are needed to account for individual variability and expectancy effects [16,19,46].

### 3.5. LSD and Pain Management

Low-dose LSD has demonstrated potential analgesic properties. In a randomized, placebo-controlled crossover study involving 24 healthy volunteers, participants received single doses of 5, 10, and 20 µg LSD, as well as placebo, on separate occasions. The 20 µg dose significantly increased pain tolerance in the Cold Pressor Test, which involved submerging a hand in cold water (3 °C), and reduced subjective ratings of pain intensity and unpleasantness. Physiological and psychological side effects—including elevations in blood pressure, dissociation, anxiety, and somatization—remained within clinically normal limits, supporting the tolerability of low-dose LSD for experimental pain modulation [47].

Although the precise neural mechanisms require further clarification, neuroimaging studies and the existing literature suggest that LSD may influence brain regions involved in sensory integration and emotional regulation, which are relevant to the processing of chronic pain [48]. These preliminary findings justify the further exploration of low-dose LSD as a potential adjunct in chronic pain management, especially in contexts where existing treatments provide insufficient relief.

### 3.6. Cognitive Flexibility, Learning, and Behavioral Therapy

LSD may enhance cognitive flexibility and learning by modulating reinforcement learning mechanisms. In a within-subjects, placebo-controlled study, participants who received 75 µg intravenous LSD demonstrated increased learning rates for both rewards and punishments during a probabilistic reversal learning task. LSD also reduced stimulus stickiness, indicating a greater tendency toward exploratory behavior rather than perseveration. These changes support the view that LSD induces a state of heightened plasticity, which may help facilitate the revision of maladaptive associations. Such effects are relevant for treating conditions characterized by cognitive rigidity, such as obsessive–compulsive disorder and major depressive disorder [49].

### 3.7. Mystical-Type Experiences and Emotional Processing

LSD’s capacity to induce mystical or transcendental experiences is considered central to its therapeutic efficacy. These experiences have been associated with long-term improvements in emotional well-being and psychological functioning [14]. Controlled studies have shown that LSD dose-dependently enhances emotional empathy, as measured by the Multifaceted Empathy Test, and increases plasma oxytocin levels, an effect attenuated by 5-HT(2A) receptor antagonism, indicating partial serotonin-mediated action [10]. Additional studies suggest that LSD promotes prosocial behavior through 5-HT(2A)/AMPA/mTORC1 signaling in the medial prefrontal cortex, a mechanism shown to be essential for social interaction enhancement in animal models [37].

Standard doses of LSD (100–200 µg) have been shown to be well tolerated in healthy participants across multiple controlled trials. Common adverse effects—such as transient increases in blood pressure, heart rate, and anxiety—were typically mild to moderate in intensity and resolved without clinical intervention [11,12]. These data support the relative safety of LSD in research settings, provided that appropriate screening and monitoring procedures are followed.

### 3.8. Co-Administration with Other Psychedelics

Psilocybin, a naturally occurring psychedelic compound found in certain mushrooms, has gained attention for its potential therapeutic effects in treating depression, anxiety, and substance use disorders. Similar to LSD, psilocybin primarily acts through serotonin (5-HT2A) receptor agonism, leading to altered perception, emotional processing, and cognitive flexibility. MDMA is a synthetic empathogen–entactogen, is known for enhancing emotional openness, empathy, and interpersonal connection, and has been studied primarily in the treatment of PTSD and other trauma-related conditions.

The co-administration of MDMA with LSD or psilocybin has been examined in a prospective study involving 698 individuals. The study found that participants who combined MDMA with psilocybin or LSD reported significantly fewer challenging experiences, including fear and grief, compared to those using psilocybin or LSD alone. Additionally, they reported greater positive emotions, such as self-compassion, love, and gratitude. However, the study found no differences in mystical-type experiences between the two groups, suggesting that MDMA may buffer against certain negative reactions without altering the depth of psychedelic effects [50].

As this study relied on self-reported data rather than controlled clinical trials, it remains uncertain whether MDMA actively reduces distress or if these effects are influenced by psychological and contextual factors. Further controlled research is necessary to determine the precise impact of MDMA co-administration on psychedelic experiences and whether it could be integrated into therapeutic protocols to enhance safety and emotional processing.

**Figure 3:**
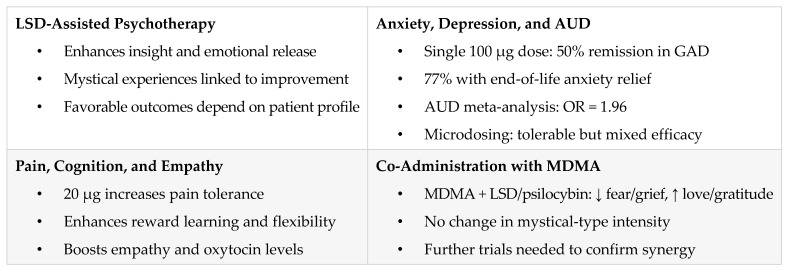
Therapeutic applications of LSD [16,41,43,44,47].

Figure 3 covers LSD’s clinical use in psychotherapy, mood disorders, AUD, microdosing, and pain, describes the emotional, cognitive, and empathogenic effects with corresponding receptor activity, and notes the emerging evidence on safety, effectiveness, and co-administration with MDMA.

Table 1 summarizes findings from various study designs exploring LSD’s therapeutic potential and clinical effects. Randomized controlled trials indicate that LSD microdosing may increase sleep duration without altering sleep stages, while high-dose LSD is well tolerated in controlled settings. Longitudinal cohort studies confirm enzyme replacement therapy (ERT) benefits in Gaucher and Pompe diseases but show limited efficacy in children with Fabry disease. Retrospective studies highlight how antidepressants modulate LSD’s effects, with most LSD-related deaths being trauma-induced. Surveys show rising LSD use, particularly among younger adults and individuals with depression, with mixed self-treatment outcomes. Meta-analyses support LSD’s long-term benefits for anxiety and alcohol use disorder, while case reports link it to persistent visual symptoms, including HPPD. Animal studies suggest that LSD may reduce alcohol consumption and enhance social behavior.

Table 2 categorizes LSD-related studies by disorder or condition, highlighting its effects across various domains. In HPPD, EEG studies indicate cortical dysregulation, with clonidine showing potential for symptom relief. LSD-assisted psychotherapy demonstrated sustained benefits for anxiety and depression, while microdosing trials for major depressive disorders are ongoing. A meta-analysis on alcohol use disorder found that a single LSD dose significantly reduced misuse, but animal studies suggest limited effects on relapse prevention. ERT improved outcomes in Gaucher and Pompe diseases but showed no significant effects in children with Fabry disease. MDMA co-administration reduced negative psychedelic experiences in PTSD. LSD also influenced cognitive flexibility, pain perception, neuroplasticity, and emotional empathy, with emerging research on public health trends and forensic toxicology.

## 4. Safety and Risk Management

### 4.1. Safety and Adverse Effects

LSD has demonstrated a favorable safety profile in controlled clinical environments, particularly when administered under professional supervision. A pooled analysis of four placebo-controlled studies involving 83 participants and 131 LSD administrations evaluated the acute effects of doses ranging from 25 to 200 µg. The findings showed that both subjective and physiological responses were dose-dependent, with positive effects—such as mood enhancement and altered perception—reported more frequently than negative effects. Physiological changes, including moderate increases in heart rate, blood pressure, and body temperature, remained within safe clinical thresholds. No serious or long-term adverse effects were observed, supporting the short-term safety of LSD in regulated research settings [18].

A separate double-blind, placebo-controlled study involving 16 healthy individuals administered 200 µg of LSD further confirmed its acute safety profile. Expected psychedelic effects were observed, including visual hallucinations, audiovisual synesthesia, and mood enhancement. Participants also reported increased interpersonal trust and openness. However, reductions in sensorimotor gating, measured via prepulse inhibition, were also noted. Physiological responses included transient elevations in heart rate, blood pressure, and hormonal levels. Importantly, no severe adverse events were recorded, reinforcing LSD’s tolerability when used under clinical supervision [33].

In contrast, data from naturalistic settings suggest an elevated risk of adverse outcomes when LSD is used without medical oversight. The 2017 Global Drug Survey, which included 10,293 respondents who reported LSD use in the previous year, found that 1.0% sought emergency medical treatment (EMT) during that period, with a per-event risk of 0.2% [60]. The most commonly reported adverse effects were psychological in nature—anxiety, panic, and confusion—often linked to unfavorable “set and setting”. The term “set” refers to an individual’s mental state, expectations, and emotional preparedness, while “setting” pertains to the social and physical environment in which the drug is used. Adverse experiences were more likely in high-stress, chaotic, or unsupported contexts. Most negative effects were resolved within 24 h, though a small subset of users (n = 11) reported symptoms persisting for over four weeks, emphasizing the importance of structured and supportive settings during psychedelic experiences.

One of the more concerning long-term risks associated with LSD use is HPPD, a condition characterized by the re-experiencing of perceptual distortions long after drug use has ceased. HPPD is generally categorized into two subtypes: Type I, involving benign or even positive recurring visual effects, and Type II, marked by distressing and functionally impairing symptoms. A clinical study with 40 participants explored differences in visual disturbances and environmental triggers among individuals with HPPD following LSD use. The most frequently reported visual disturbance among Type I participants was the slow movement of still objects, whereas trailing phenomena were more common in Type II. Distinct triggers also emerged: individuals with Type I more often reported disturbances in dark environments, during sexual activity, or while focusing on static or moving objects [59].

While the study did not quantify the distribution of each subtype, the previous literature suggests that Type I is more prevalent than Type II. Nonetheless, the small sample size and lack of epidemiological data leave the true prevalence of HPPD unclear. These findings indicate the need for further investigation into the etiology and management of HPPD. Clonidine, an α2-adrenergic agonist commonly used to treat hypertension and symptoms of sympathetic overactivity, has shown promise in managing HPPD. In an open-label pilot study involving eight patients who had experienced LSD-induced HPPD for at least three months, low-dose clonidine (0.025 mg three times daily) was administered over a two-month period [58]. Baseline assessments indicated marked psychopathology, with average scores of 5.25 on the Clinical Global Impression Scale (CGI) and 4 on a self-reported symptom severity scale. By the end of the trial, six participants remained, with average scores improving to 2.5 (CGI) and 2 (self-report), indicating a reduction to mild symptom levels. The results suggest that clonidine may alleviate LSD-related flashbacks linked to excessive sympathetic nervous activity.

LSD may pose greater risks for individuals with pre-existing psychiatric vulnerabilities. Although rare, psychological distress and complications can occur, particularly in unsupervised or unstructured settings. Large-scale analyses suggest that while LSD is not inherently life-threatening, its use may lead to significant psychological discomfort in susceptible individuals, highlighting the importance of harm-reduction strategies and mental health screening prior to administration [75].

Recent clinical trials have emphasized the protective role of a controlled environment in mitigating these risks. Structured clinical settings that include preparatory and integrative psychological support have been shown to enhance safety and improve therapeutic outcomes, further reinforcing the importance of context in psychedelic research and therapy [77].

### 4.2. Pharmacological Interactions and Variability in Safety

LSD’s pharmacological effects can be significantly influenced by concurrent medication use, particularly in individuals undergoing treatment with psychiatric medications. Evidence suggests that the chronic administration of certain antidepressants modulates LSD’s psychoactive effects. For example, tricyclic antidepressants and lithium have been associated with enhanced subjective responses to LSD, whereas monoamine oxidase inhibitors (MAOIs) appear to reduce its effects [9]. These interactions highlight the importance of careful medication screening prior to LSD administration in both research and therapeutic contexts, as such pharmacodynamic interactions may alter efficacy, increase unpredictability, or elevate the risk of adverse effects.

Beyond pharmacological interactions, individual variability in LSD’s behavioral effects may be partially explained by receptor- and circuit-specific mechanisms. Preclinical studies have shown that LSD enhances social behavior via serotonin 5-HT_2_A receptor activation and downstream AMPA receptor and mTORC1 signaling within excitatory neurons of the medial prefrontal cortex (mPFC). In genetically modified mouse models, LSD failed to induce prosocial behavior in mice lacking Raptor—a key component of mTORC1—in excitatory neurons. In contrast, this effect was preserved in mice lacking Raptor in GABAergic neurons [37]. These findings suggest that differences in excitatory neuronal signaling pathways could underlie individual variability in behavioral outcomes following LSD administration. Such variability may have important safety implications, particularly for individuals with neuropsychiatric disorders or altered neural circuitry.

### 4.3. Analytical and Forensic Detection

The accurate detection and quantification of LSD and its metabolites are essential for safety monitoring, forensic toxicology, and compliance with controlled substance regulations. Advanced analytical techniques, such as liquid chromatography–tandem mass spectrometry (LC-MS/MS), have been validated for the detection of LSD and its primary metabolites—including iso-LSD, nor-LSD, and 2-oxo-3-hydroxy-LSD—in biological matrices such as plasma, hair, and urine. These methods achieve limits of quantification as low as 0.05 ng/mL, providing high sensitivity and specificity for forensic and clinical applications [26,27,72].

Sample stability is another critical consideration in forensic toxicology. Studies have demonstrated that fluoride-treated biological samples inhibit the degradation and metabolic conversion of LSD and its analogs, particularly N1-substituted compounds, thereby preserving sample integrity for accurate analysis [27]. These findings are especially important for the detection of designer lysergamides and for monitoring compliance in clinical trials.

In parallel, neuroimaging techniques have contributed to the understanding of LSD’s central nervous system effects. Positron emission tomography (PET) studies have mapped LSD binding sites across the brain, revealing high-density occupancy in regions such as the frontoparietal cortex, anterior cingulate cortex, nucleus accumbens, and dentate gyrus [32]. These imaging studies support the development of in vivo biomarkers for monitoring the neuropharmacological effects of LSD in both research and potential therapeutic applications.

Taken together, advancements in analytical chemistry and neuroimaging have significantly improved the ability to characterize LSD’s pharmacokinetics, receptor activity, and physiological effects. LSD’s strong affinity for the 5-HT_2_A receptor and secondary activity at dopaminergic and adrenergic sites contribute to a complex pharmacological profile. Physiological effects—such as increased cardiovascular activity and hormonal fluctuations—reinforce the necessity for controlled administration, especially in individuals with underlying medical conditions.

### 4.4. Risk Management

While LSD has demonstrated a favorable safety profile in controlled clinical settings [18], risk management remains a critical consideration, particularly in unsupervised or recreational contexts. Adverse effects such as hallucinations, agitation, panic, and, in rare cases, the need for emergency medical treatment have been reported, especially when LSD is used in environments lacking appropriate psychological and medical support [28,60,75].

A specific concern in the long-term safety profile of LSD is the development of persistent visual disturbances, notably HPPD. This condition is characterized by enduring perceptual anomalies such as visual trails or afterimages. Multiple case studies and reviews have emphasized the need for further research into the etiology and prevalence of HPPD, particularly in individuals with repeated or high-dose use [58,59,66].

Individual variability in response to LSD further complicates risk assessment. Microdosing trials have reported anxiety-related participant withdrawals, highlighting that even low doses can produce adverse psychological responses in some individuals [19]. At higher doses, LSD is known to induce profound alterations in consciousness, including mystical-type experiences and perceptual distortions, which may be disorienting or overwhelming without proper support [14,33]. These findings underscore the importance of dose titration and individualized treatment protocols.

To mitigate risks, safety-focused studies should continue to investigate potential harms associated with self-harm, accidents, and psychiatric destabilization—particularly in nonclinical use contexts [28,60]. Structured harm-reduction strategies have been proposed and increasingly implemented in clinical trials and therapeutic programs. These include thorough psychological preparation prior to LSD administration, ongoing monitoring by trained facilitators, and post-session integration therapy aimed at helping individuals process and contextualize their experiences [14,41,60].

Long-term follow-up studies are also needed to assess the incidence and progression of persistent perceptual disturbances such as HPPD, especially among users who engage in repeated or high-dose use [58,59,66]. Understanding the risk factors and potential preventive measures for these conditions is essential for ensuring the safety of both clinical participants and broader user populations.

Pharmacological strategies to manage adverse effects have also been explored. Risperidone, a serotonin 5-HT_2_A and dopamine receptor antagonist, has shown potential in animal models for counteracting LSD’s psychoactive effects [73]. Although further research is needed to validate its efficacy in humans, such interventions may offer an additional safeguard in therapeutic settings where acute symptom management is required.

**Figure 4:**
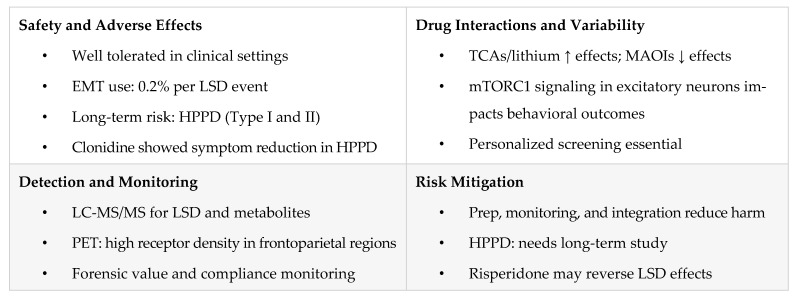
Safety and risk management of LSD [18,33,58,59,60].

Figure 4 outlines LSD’s safety profile in clinical vs. nonclinical settings, highlights HPPD subtypes, clonidine intervention, emergency treatment rates, and interaction risks, and stresses the importance of harm reduction, screening, and controlled settings.

Table 3 summarizes the safety profile of LSD, including its acute effects, risks, and potential long-term consequences. Clinical trials indicate that LSD is generally well tolerated, with dose-dependent increases in heart rate and blood pressure, but no severe or long-term adverse effects have been observed. Common adverse effects include anxiety, panic, confusion, and agitation, often influenced by the user’s mindset and environment (set and setting). Severe reactions, such as hyperthermia, seizures, and cardiovascular issues, are rare and more common in polysubstance use cases. LSD does not induce compulsive drug-seeking behavior, and no physical dependence or withdrawal symptoms have been reported. However, some users experience persistent visual disturbances (HPPD, palinopsia), while individuals with psychiatric conditions may be at higher risk for prolonged psychosis-like symptoms.

## 5. Public Health and Epidemiology

### 5.1. Population and Use Trends

Recent epidemiological data reveal significant shifts in LSD use patterns, particularly among populations with mental health challenges and younger adults. An analysis of U.S. national survey data from 2008 to 2019 found a marked increase in past-year LSD use among individuals with major depressive disorder compared to those without depression. The most pronounced increases were observed among adults aged 18–34 and individuals with lower household incomes, suggesting a possible trend of self-directed use for mental health purposes [62]. These findings underscore the growing public interest in psychedelics as mental health interventions and highlight the need for targeted harm-reduction strategies to address unsupervised use.

A separate analysis covering the period from 2015 to 2018 reported a 56.4% increase in past-year LSD use among U.S. adults [63]. The most significant rises were among individuals aged 26–49, those with higher education levels, and bisexual individuals. Conversely, LSD use declined among those with lower educational attainment and those perceiving higher risk associated with psychedelic use. These demographic shifts may reflect evolving societal attitudes, increased accessibility, and broader cultural acceptance of psychedelics. Taken together, these trends suggest an urgent need for public health responses to adapt through updated prevention frameworks and risk-mitigation policies that address the changing landscape of LSD use.

### 5.2. Public Health and Demographic

The increasing prevalence of LSD use among individuals with depression suggests a growing reliance on psychedelics for self-medication, reinforcing the importance of conducting well-regulated clinical trials to assess safety, efficacy, and appropriate therapeutic contexts [62]. Broader demographic shifts in use patterns—such as increased use among highly educated individuals and those identifying as bisexual—further signal rising societal acceptance and changing perceptions of psychedelics [63].

Emerging research into LSD’s pharmacological profile supports this growing interest, showing its capacity to enhance neuroplasticity. Specifically, LSD has been found to increase the levels of brain-derived neurotrophic factor (BDNF), a molecule essential for synaptic plasticity and neural regeneration [36]. These effects suggest potential therapeutic applications in treating conditions like depression and other neuropsychiatric disorders. Additionally, LSD may enhance cognitive flexibility and learning adaptability, indicating potential utility in improving executive function and reducing maladaptive behaviors such as addiction [49].

As these mechanisms align with promising psychiatric applications, public health initiatives should focus on bridging the gap between scientific findings and community use. This includes promoting safe, informed, and equitable access to psychedelic care under appropriate medical supervision.

### 5.3. Nonmedical Use and Associated Risk

While interest in LSD’s therapeutic potential continues to grow, its rising nonmedical use presents notable public health concerns. Increases in use have been particularly observed among young adults with major depression [62], bisexual individuals, and those with higher education levels [63]. These trends raise concerns about unsupervised self-treatment, especially in the absence of clinical oversight.

Adverse psychological effects—including panic, confusion, and distressing experiences—have been reported in nonclinical contexts. Although emergency medical interventions related to LSD use remain relatively rare, they nonetheless highlight the importance of harm-reduction strategies [60,61]. Persistent psychological effects or prolonged distress can occur, particularly in users lacking adequate preparation, support, or knowledge.

To mitigate these risks, future research should explore the motivations behind nonmedical LSD use and the psychosocial factors contributing to its rise. Expanding access to supervised psychedelic-assisted therapy, alongside comprehensive public education initiatives, may help reduce the risks associated with self-medication and encourage safer practices [75]. Public health responses should prioritize harm reduction, informed consent, and the development of resources that equip individuals with accurate information and access to professional support—essential steps for balancing the therapeutic promise of LSD with the realities of its use in broader, real-world settings.

**Figure 5:**
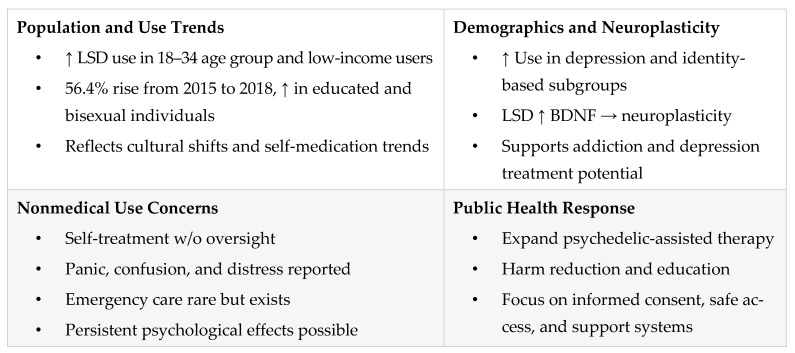
Public health and epidemiology of LSD use [36,60,62,63,75].

Figure 5 presents population-level trends in LSD use, emphasizing increases among younger adults and self-treatment motivations, identifies nonmedical use risks, and advocates for public education, supervised therapy access, and harm-reduction strategies.

## 6. Research Challenges and Methodological Gaps

LSD research spans a diverse array of disciplines, including psychiatry, neuroscience, pharmacology, and behavioral science. Despite notable progress, clinical and preclinical investigations continue to face significant methodological, regulatory, and translational challenges. These limitations hinder the generalizability of findings and the development of standardized therapeutic protocols.

### 6.1. Sample Size and Participant Bias

One of the most common limitations in LSD research is the reliance on small sample sizes, which restrict statistical power and reduce the generalizability of findings. Many studies to date have been exploratory or pilot trials, often involving fewer than 50 participants [10,16,17,18,36,49,70,76]. Additionally, the majority of trials have been conducted in healthy volunteers rather than clinical populations, such as individuals with depression, anxiety disorders, or chronic pain [16,17,19,22,43,47,78]. Vulnerable groups—including those with comorbid psychiatric or substance use disorders—remain underrepresented, limiting the clinical applicability of research outcomes [18,36,43,49].

### 6.2. Dosage, Context, and Study Design Variability

Considerable variability in dosing regimens across studies presents another major obstacle to interpreting and comparing findings. Protocols range from microdosing (≤20 µg) to full-dose psychedelic therapy (100–200 µg), with investigations conducted in both controlled laboratory environments and naturalistic settings [15,19,44,46,70,76,78]. Moreover, LSD’s effects are highly context-dependent, influenced by psychological and environmental variables often referred to as “set and setting”. While several studies have acknowledged the role of these factors in shaping mystical-type experiences and subjective outcomes [13,14,41,76], there remains a lack of systematic research into how context interacts with LSD’s pharmacological effects. Most studies have focused on acute outcomes such as emotional modulation, cognitive flexibility, and brain connectivity [17,20,21,22,26]. However, the long-term persistence of these effects is not well understood. Some research has begun to evaluate sustained outcomes beyond the acute phase [19,46,47,78], but more longitudinal studies are needed to determine the durability of therapeutic benefits.

### 6.3. Limited Long-Term Data

Short-term reductions in symptoms of anxiety and depression have been documented in several trials [16,42], as well as reductions in alcohol misuse following single-dose LSD administration [44,67]. However, there is limited evidence regarding the long-term efficacy and safety of LSD, particularly with repeated use in clinical populations such as those with major depressive disorder (MDD) [16,46]. Furthermore, the prevalence and outcomes of self-treatment practices among nonmedical users remain underexplored, complicating risk assessment in real-world settings [62]. Persistent adverse effects—most notably HPPD—raise concerns regarding the potential risks associated with high-dose or chronic LSD use [58,59,66]. The mechanisms and prevalence of HPPD remain unclear, necessitating further research into its etiology, diagnosis, and treatment.

### 6.4. Gaps in Mechanistic Understanding

Although LSD’s activity at serotonin 5-HT_2_A and dopamine receptors is well established, the relationship between receptor-level activity and clinical outcomes remains incompletely understood. Neurobiological findings—such as increased global brain connectivity [11,12] and elevated levels of brain-derived neurotrophic factor (BDNF) [36]—suggest mechanisms underlying cognitive flexibility and emotional processing. However, these effects have yet to be unified into a comprehensive model that explains both therapeutic efficacy and potential adverse effects [35,49,65]. Receptor binding studies using radiolabeled ligands ([^125^I]LSD and [^125^I]DOI) have contributed to our understanding of LSD’s distribution in the brain [32], but these findings have not been fully integrated into pharmacogenetic or outcome-based models. Further investigation is required to determine how individual variability in receptor expression and signaling affects treatment response.

### 6.5. Lack of Integration with Pharmacogenetics

Pharmacogenetic factors—including polymorphisms in serotonin receptor genes and cytochrome P450 enzymes (CYP2D6, CYP2E1, CYP3A4)—may play a significant role in modulating individual responses to LSD [9,23]. Despite this, pharmacogenetic influences on LSD’s efficacy and tolerability have received minimal attention in clinical research. Understanding genetic variability could facilitate the development of personalized psychedelic therapies, allowing clinicians to predict both therapeutic response and susceptibility to adverse reactions [23]. The systematic integration of pharmacogenetic screening into clinical trials could enhance the safety and precision of LSD-assisted interventions.

### 6.6. Preclinical Clinical Translation Issues

Although preclinical models provide valuable mechanistic insights, translating these findings into human contexts remains challenging. Animal studies have elucidated LSD’s behavioral and neuroplastic effects [37,45,69], yet models of psychiatric conditions such as anxiety, depression, and addiction often lack translational fidelity due to differences in emotional and cognitive complexity [45]. Additionally, while novel lysergamide analogs such as 1B-LSD, 1CP-LSD, and 1DD-LSD have been structurally characterized and tested in preclinical systems, their pharmacological activity in humans remains largely unverified [29,34,68]. Without robust translational data, it is difficult to determine the therapeutic or safety implications of these compounds.

### 6.7. Regulatory and Ethical Constraints

Regulatory barriers remain a primary limitation in the advancement of LSD research. As a Schedule I substance, LSD is subject to strict legal controls that hinder funding, participant recruitment, and the execution of large-scale, multi-site clinical trials [7,8,43]. In addition, the historical stigma associated with LSD—particularly its association with countercultural movements and nonmedical use—continues to influence public policy and research priorities. Although scientific interest in LSD has grown, legal and institutional constraints persist, limiting the expansion of clinical investigations and delaying integration into mainstream psychiatric care [7,8].

**Figure 6:**
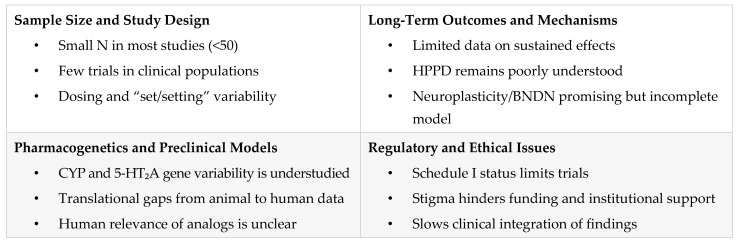
Research challenges and methodological gaps [7,16,23,29,34,35,36,44,58].

Figure 6 highlights the limitations in LSD research, including small sample sizes, inconsistent dosing, a lack of long-term data, and poor preclinical translation, and stresses the need for pharmacogenetics, mechanistic clarity, and regulatory reform.

## 7. Future Directions and Innovations in LSD Research

As interest in LSD’s therapeutic potential continues to grow, future research must address current methodological gaps and expand into new domains. This includes improving clinical trial design, investigating long-term outcomes, exploring mechanistic underpinnings, and developing personalized approaches through pharmacogenetics. Advancements in neuroscience and regulatory reform will also be essential to the safe and effective integration of LSD into therapeutic contexts.

### 7.1. Larger, Inclusive Clinical Trials

Many existing LSD studies are limited by small sample sizes and predominantly involve healthy participants, restricting the generalizability of findings [10,15,16,17,43]. Larger randomized controlled trials (RCTs) involving diverse and clinically relevant populations—such as individuals with treatment-resistant depression, PTSD, or substance use disorders—are essential to better evaluate safety, efficacy, and clinical applicability [16,46]. Designing trials with robust statistical methodologies, such as Fisher’s LSD procedure, Bonferroni corrections, or Hochberg procedures, can improve analytical precision and accommodate complex comparisons [79]. Ongoing placebo-controlled studies on LSD microdosing are expected to yield important data on its cognitive, emotional, and functional outcomes [78]. Future trials should prioritize conditions such as major depressive disorder (MDD) [16], AUD [44,67], and PTSD [41]. Emerging evidence also suggests potential applications in social dysfunction, with studies pointing to LSD’s role in enhancing empathy and prosocial behavior through oxytocin modulation [10,37].

### 7.2. Standardization of Protocols and Dosing

One major challenge in LSD research is the variability in dosing regimens and study designs. Current investigations range from microdoses (≤20 µg) to full psychedelic doses (100–200 µg), making cross-study comparisons difficult [17,18,22,44]. Standardized dose–response protocols are necessary for both microdosing [46,78] and full-dose applications. Additionally, consistent consideration of non-pharmacological influences—namely “set and setting”—is essential for improving study reliability and clinical translation [14,41,76]. Analytical techniques such as LC-MS/MS should be routinely incorporated to quantify LSD and its metabolites, enhancing pharmacokinetic assessments [26,27].

### 7.3. Long-Term and Longitudinal Research

The majority of LSD research focuses on acute effects, leaving gaps in understanding its long-term safety and efficacy. Longitudinal studies are needed to assess the durability of symptom relief, the potential benefits of repeated dosing, and the emergence of long-term adverse effects, such as HPPD [58,59,66]. Microdosing trials should investigate whether therapeutic effects persist over extended periods and whether repeated low-dose use remains safe in conditions like MDD, PTSD, and addiction [16,19,46,47].

### 7.4. Mechanistic Research and Brain Modeling

Further research is needed to elucidate LSD’s mechanisms of action. Advanced neuroimaging tools such as fMRI and EEG should be employed to explore its effects on large-scale brain networks, including the default mode network (DMN) and cortico-striato-thalamo-cortical (CSTC) circuits [11,12,48,70]. Understanding the role of serotonin (5-HT_2_A) and dopamine receptor activity is also key to decoding LSD’s influence on mood, perception, and cognition [31,49,65]. Neuroplasticity markers such as BDNF may underlie long-term behavioral and cognitive changes [36,65]. LSD’s analgesic properties, shown in studies demonstrating increased pain tolerance without full psychedelic effects, warrant further exploration for chronic pain management [47]. Additionally, early data suggest that LSD may be well tolerated in older adults, highlighting its potential relevance in neurodegenerative conditions [52].

### 7.5. Advanced Neuroscientific Approaches: Brain Dynamics and Predictive Models

Advancements in neuroimaging and computational neuroscience have shed light on LSD’s impact on brain dynamics. Studies using fMRI and dynamic causal modeling reveal that LSD disrupts equilibrium states and increases neural sensitivity to external stimuli [70]. These alterations may explain its therapeutic potential in disorders marked by cognitive rigidity, such as PTSD and depression. Computational models and machine learning approaches could help predict individual responses to LSD therapy, aiding in treatment planning and risk mitigation.

### 7.6. Integration of Pharmacogenetics and Personalized Medicine

Genetic variations in serotonin receptor function and cytochrome P450 enzymes (CYP2D6, CYP2E1, CYP3A4) may significantly influence LSD’s pharmacokinetics and pharmacodynamics [9,23]. Future research should incorporate pharmacogenetic analyses to identify genetic markers that predict therapeutic response or adverse reactions. A deeper understanding of genetic variability in LSD metabolism and receptor binding may support the development of personalized treatment approaches [12,23].

### 7.7. Novel LSD Analogs

Emerging lysergamides such as 1B-LSD, 1CP-LSD, and 1DD-LSD require further study to evaluate their pharmacological profiles and clinical relevance. 1CP-LSD, identified as a prodrug for LSD, has been shown to convert to active LSD in human serum, though further pharmacokinetic data are needed [29]. 1DD-LSD exhibits significantly reduced potency in animal models, but its distinct psychoactive profile has not yet been fully characterized [34]. Comparative studies on these analogs may reveal alternative therapeutic pathways or safety advantages over traditional LSD.

### 7.8. LSD Co-Use with Other Substances

Preliminary survey data suggest that combining LSD or psilocybin with MDMA may reduce challenging experiences (e.g., fear, grief) while enhancing positive emotional states such as self-compassion and gratitude. However, these findings are based on self-reported data and require validation through controlled clinical trials. Rigorous investigation is needed to determine the safety, efficacy, and potential therapeutic utility of co-administration protocols [50].

### 7.9. Cross-Disciplinary Collaboration

Interdisciplinary research that integrates pharmacology, neuroscience, psychopharmacology, and behavioral science can enhance our understanding of LSD’s complex effects [10,11,12]. Collaborations with forensic toxicology and public health fields will be essential for monitoring drug trends, developing safety guidelines, and informing policy [26,27].

### 7.10. Regulatory Advocacy and Public Education

LSD’s classification as a Schedule I substance continues to hinder large-scale research and clinical translation. Regulatory advocacy aimed at reclassification could enable broader scientific inquiry and therapeutic development [7,8]. Public education initiatives are also needed to disseminate balanced, evidence-based information about LSD’s risks and potential benefits. Such efforts can inform clinicians, researchers, and the public, supporting safer use and more informed decision-making [75].

**Figure 7:**
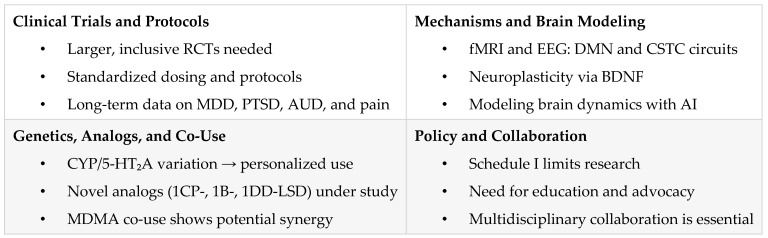
Future directions and innovations in LSD research [8,10,11,16,23,29,36,46,50].

Figure 7 outlines research priorities, including larger trials, standardization, long-term outcomes, brain modeling, personalized approaches, analog evaluation, and co-use protocols, and advocates for cross-disciplinary collaboration and regulatory change to expand clinical and scientific access.

Table 4 outlines key areas of ongoing and future research into LSD’s potential therapeutic applications. Neuroplasticity studies suggest LSD enhances cognitive flexibility and increases BDNF levels, indicating possible benefits for cognitive and neuropsychiatric disorders. Research on pain management has found that LSD modulates pain-processing brain regions and increases pain tolerance, supporting its potential as an analgesic. LSD-assisted therapy has shown promise for anxiety, alcohol use disorder, and depression, but further research is needed to optimize protocols. Microdosing studies indicate transient mood-enhancing effects but no long-term cognitive benefits. While LSD’s effects on brain networks and serotonin activity suggest potential in neurological disorders, no direct evidence supports its use in Alzheimer’s or Parkinson’s disease. Social behavior research highlights LSD’s potential role in empathy enhancement, though more studies are required.

## 8. Conclusions

LSD represents a promising avenue in psychiatric and neurological research, offering unique therapeutic potential through its profound effects on brain connectivity, neuroplasticity, and cognition. However, progress is hindered by methodological inconsistencies, limited long-term safety data, and regulatory barriers. To fully realize LSD’s clinical potential, future research must prioritize large-scale, diverse clinical trials with standardized protocols and robust safety monitoring. Advancing pharmacogenetic research and investigating novel LSD analogs could pave the way for personalized medicine approaches. By addressing these challenges through interdisciplinary collaboration and public education, LSD may transition from a stigmatized substance to an evidence-based therapeutic option for treatment-resistant conditions and unmet medical needs.

## Figures and Tables

**Table 1:** Clinical evidence and therapeutic potential.

Study Type	Agents Used and Doses	Key Findings	References
Randomized controlled trials (RCTs)	- LSD microdosing (10 μg every third day) - High-dose LSD (50–200 μg) - MEVAC^®^ vaccine for lumpy skin disease (LSDV) - Ongoing Phase 2b trial (LSDDEP2) for major depressive disorder	- LSD microdosing increased sleep duration but had no effect on sleep stages or physical activity. - High-dose LSD was well tolerated in controlled settings, with effects lasting up to 12 h and no significant long-term adverse effects. - MEVAC^®^ vaccine was safe and effective in cattle. - No clinical outcomes reported yet from LSDDEP2 trial.	[15,17,18,46,48,51,52]
Longitudinal cohort studies	- Enzyme replacement therapy (ERT) for Gaucher, Pompe, and Fabry diseases	- Improved blood markers and mobility in Gaucher and Pompe diseases. - In Fabry disease, ERT reduced left ventricular mass index and proteinuria risk in adults but had no significant effects in children.	[53,54,55,56]
Retrospective studies	- LSD interactions with antidepressants - LSD-related deaths - Historical LSD psychotherapy - HPPD cases	- Tricyclic antidepressants and lithium increased LSD effects, while monoamine oxidase inhibitors reduced them. - Most LSD-related deaths were trauma-related, with self-harm cases involving physical means. - Historical LSD psychotherapy had mixed outcomes—some short-term improvements but poor long-term results. - HPPD symptoms persisted for years in some cases.	[7,9,28,57,58,59]
Cross-sectional studies/surveys	- General population LSD use trends - Self-treatment with psychedelics - MDMA co-administration with LSD/psilocybin	- LSD use increased, particularly among individuals with depression and younger adults. - Self-treatment was common, with both positive (improved mood, insight) and negative effects (anxiety, distress). - MDMA co-administration reduced negative experiences (e.g., fear, grief) during psychedelic use.	[50,60,61,62,63]
Meta-analyses/systematic reviews	- LSD for anxiety in life-threatening illnesses - LSD for alcohol use disorder - LSD dose–response - Comparative efficacy of psychedelics (LSD, psilocybin, MDMA, ketamine)	- LSD showed long-term benefits for anxiety in life-threatening illnesses. - Significantly improved alcohol use disorder outcomes. - Effects plateaued at ~100 µg (higher doses do not necessarily enhance effects). - Distinct therapeutic potentials identified among LSD, psilocybin, MDMA, and ketamine.	[13,43,44,64,65]
Case series/case reports	- LSD-induced visual disturbances (HPPD, palinopsia) - Clonidine for HPPD treatment	- HPPD symptoms persisted in some LSD users. - Clonidine reduced HPPD symptoms in certain cases.	[58,66]
Animal studies	- LSD (50 μg/kg) in mice - LSD analogs (1P-ETH-LAD, 1CP-LSD) - LSD in rats (behavioral effects) - LSD-induced social behaviors in mice	- LSD reduced alcohol consumption in mice. - Certain LSD analogs functioned as prodrugs, converting to LSD or ETH-LAD in vivo. - LSD transiently increased anxiety-like behaviors in rats. - LSD promoted social behaviors via 5-HT_2_A and AMPA receptor activation.	[29,37,67,68,69]
Pharmacokinetic/pharmacodynamic studies	- LSD (5–200 μg) - LSD BDNF effects (5–20 μg)	- First-order elimination kinetics with peak plasma concentrations at ~1.4–1.7 h. - Subjective effects lasted 8–12 h. - Low doses (5–20 μg) increased BDNF levels, suggesting neuroplasticity effects.	[10,20,21,22,26,36]
Neuroimaging/fMRI studies	- LSD effects on brain connectivity - LSD and pain perception - LSD and emotional empathy	- LSD increased whole-brain connectivity while reducing local coherence in specific regions. - Altered pain perception by reducing activity in pain-processing areas. - Enhanced emotional empathy, correlating with thalamic activity and changes in the default mode network (DMN).	[11,12,33,35,48,49,70]
Computational/modeling studies	- LSD-induced brain dynamics - Machine learning and LSD - LSD1 enzyme inhibition (cancer research)	- LSD shifted brain dynamics further from equilibrium, increasing response flexibility. - Machine learning identified LSD-induced connectivity changes with 91.11% accuracy. - Computational models were developed for LSD1 enzyme inhibition, targeting potential cancer therapies.	[32,35,71]
Analytical/forensic studies	- LSD analog detection (ETH-LAD, 1P-ETH-LAD, 1CP-LSD) - LSD stability in biological samples	- Novel LSD analogs identified, with some acting as prodrugs. - New analytical methods improved LSD detection in hair, urine, and plasma. - Sodium fluoride (NaF) storage minimized LSD degradation in biological samples.	[27,29,30,72]
Psychopharmacology/cognitive studies	- LSD and cognitive flexibility - LSD and reinforcement learning - LSD and pain perception - LSD receptor interactions (serotonin-mediated)	- LSD increased cognitive flexibility and altered reinforcement learning by enhancing learning rates for both reward and punishment. - LSD’s effects were mediated primarily by serotonin receptors. - Risperidone effectively blocked LSD’s effects. - LSD reduced pain perception in controlled settings.	[47,49,73]

**Table 2:** Studies categorized by type of disorder.

Disorder/Condition	Key Findings	References
Hallucinogen-persisting perception disorder (HPPD)	EEG studies in HPPD patients showed widespread reduced cortical coherence in the eyes-open state and increased occipital coherence upon eye closure, suggesting visual cortex dysregulation. Clonidine treatment reduced HPPD symptoms in some patients. LSD-induced palinopsia persisted for years in rare cases.	[58,66,74]
Mood disorders (depression, anxiety, seasonal affective disorder—SAD)	LSD-assisted psychotherapy provided sustained benefits for anxiety in life-threatening illnesses and showed potential in depression treatment. A Phase 2b trial (LSDDEP2) is ongoing to evaluate LSD microdosing for major depressive disorder, but clinical outcomes have not yet been reported. Bright light therapy for seasonal affective disorder (SAD) was correlated with changes in serotonin receptor binding.	[16,38,43,46,65]
Substance use disorders (alcoholism, addiction, relapse prevention)	A meta-analysis showed that a single LSD dose significantly reduced alcohol misuse in clinical settings. In animal studies, LSD reduced alcohol consumption in mice at a 50 μg/kg dose but did not prevent relapses in addiction models.	[44,45,67]
Neurodegenerative/neuromuscular disorders (Gaucher, Fabry, Pompe, MPS, NPC)	Long-term enzyme replacement therapy (ERT) improved platelet counts, hemoglobin levels, organ function, and mobility in Gaucher and Pompe diseases. In Fabry disease, ERT provided benefits in adults (e.g., reduced left ventricular mass index and proteinuria risk) but had no significant effects in children.	[53,54,55,56]
Pain disorders (chronic pain, analgesia, migraine)	LSD reduced activity in pain-processing brain regions, including the anterior cingulate cortex, thalamus, and insula. A low dose of LSD (20 μg) increased pain tolerance in a Cold Pressor Test without inducing a full psychedelic experience.	[47,48]
Psychosis/schizophrenia	LSD-displacing factors in cerebrospinal fluid (CSF) were elevated in unmedicated psychotic patients and correlated with symptom improvement after antipsychotic treatment. LSD’s effects were blocked by risperidone, confirming its interaction with serotonin and dopamine receptors.	[40,73]
Cognitive/executive functioning disorders	LSD increased cognitive flexibility, improved reinforcement learning rates for rewards and punishments, and reduced stimulus stickiness, indicating heightened exploration. These findings suggest potential therapeutic applications for cognitive disorders.	[49]
Cardiovascular/autonomic disorders	LSD binding was highest in fetal brainstem regions involved in cardiovascular and respiratory regulation. A small number of LSD-related cardiovascular deaths were reported, but most fatalities were trauma-related.	[28,39]
Forensic/toxicology Studies (LSD-related deaths, emergency treatment, poisoning)	LSD-related deaths were primarily trauma-induced, with a low risk of acute toxicity. LSD cases resulted in more hospital admissions than psilocybin cases. New forensic detection methods improved LSD identification in biological samples, with sodium fluoride storage minimizing LSD degradation.	[27,28,60,61,75]
Psychopharmacology/neuroplasticity	LSD increased brain-derived neurotrophic factor (BDNF) levels, suggesting potential neuroplasticity effects. Music significantly influenced LSD-induced brain dynamics, highlighting its role in psychedelic therapy settings.	[36,76]
Public health and LSD usage trends	LSD use increased significantly among young adults, individuals with depression, and those with higher education. Changing social attitudes and increased accessibility were identified as potential factors influencing usage patterns.	[62,63]

**Table 3:** LSD’s safety profile.

Category	Key Findings	References
Acute safety profile	LSD was generally well tolerated in controlled settings, producing dose-dependent increases in heart rate, blood pressure, and transient psychological effects. No severe or long-term adverse effects were observed in controlled trials.	[17,18]
Adverse effects and risks	The most commonly reported adverse effects of LSD include anxiety, panic, confusion, and agitation, often influenced by set and setting. Severe effects, such as hyperthermia, seizures, and cardiovascular complications, were rare but more likely in cases involving polysubstance use.	[28,60,61,75]
Emergency medical treatment (EMT) risk	Approximately 1% of LSD users sought emergency medical treatment (EMT) within a year, with a per-event risk of 0.2%. Psychological distress, including anxiety and panic, was the most common reason for hospital visits.	[60]
HPPD and visual disturbances	Persistent visual disturbances, including HPPD and palinopsia, have been reported in some LSD users, sometimes lasting for years. EEG studies in HPPD patients suggest reduced cortical coherence in the eyes-open state and increased occipital coherence upon eye closure, indicating cortical dysregulation. Clonidine has shown potential in reducing HPPD symptoms in some patients.	[58,66,74]
Potential for addiction or dependence	LSD does not produce compulsive drug-seeking behavior typical of addictive substances. No evidence of physical dependence or withdrawal symptoms has been reported in controlled studies.	[18]
Psychological risks	While some individuals experience profound positive effects, LSD may cause transient anxiety or psychological distress, particularly in those with underlying psychiatric conditions. Individuals with pre-existing psychiatric disorders may be at increased risk for prolonged or severe adverse psychological reactions. Further research is needed to determine LSD’s effects on psychosis vulnerability.	[65,73]

**Table 4:** LSD’s future research directions.

Research Area	Key Findings	References
Neuroplasticity and cognitive effects	LSD enhances cognitive flexibility and may promote brain plasticity by increasing BDNF levels. These effects suggest potential applications in cognitive and neuropsychiatric disorders.	[36,49]
LSD and pain management	LSD altered pain perception by reducing activity in pain-processing brain regions, including the anterior cingulate cortex, thalamus, and insula. A low dose of LSD (20 μg) increased pain tolerance in a Cold Pressor Test, supporting its potential as an analgesic.	[47,48]
LSD and therapy	Clinical trials suggest that LSD-assisted therapy may offer long-term benefits for anxiety in life-threatening illnesses, alcohol use disorder, and depression. Further research is needed to optimize treatment protocols.	[43,44,65]
Emerging research: LSD microdosing	Ongoing studies are investigating whether LSD microdosing can improve mood, creativity, and cognition. Preliminary findings suggest transient mood-enhancing effects but no significant long-term changes in overall mood or cognition.	[19,46,78]
Emerging research: psychedelics in neurological disorders	LSD’s effects on brain networks, neuroplasticity, and serotonin receptor activity suggest potential therapeutic applications for neurological disorders. However, no direct evidence currently supports its use in neurodegenerative conditions such as Parkinson’s or Alzheimer’s. Further research is needed.	[36,39]
Interdisciplinary research: psychedelics and social behavior	LSD has been shown to enhance social behavior in animals and increase emotional empathy in humans. These effects are mediated through serotonin 5-HT_2_A and AMPA receptor activation. Further research is needed to determine its potential therapeutic applications.	[10,37]

## Data Availability

No new data were created or analyzed in this study. Data sharing is not applicable to this article.
